# A Herbal Formula, *Atofreellage*, Ameliorates Atopic Dermatitis-Like Skin Lesions in an NC/Nga Mouse Model

**DOI:** 10.3390/molecules21010035

**Published:** 2015-12-25

**Authors:** Won-Yong Kim, Hyeong-Geug Kim, Hye-Won Lee, Jin-Seok Lee, Hwi-Jin Im, Hyo-Seon Kim, Sung-Bae Lee, Chang-Gue Son

**Affiliations:** 1 Liver and Immunology Research-Center, Daejeon Oriental Hospital of Daejeon University, 176-9, Daeheung-ro, Jung-Gu, Daejeon 34929, Korea; godunga3@naver.com (W.-Y.K.); winakim@dju.kr (H.-G.K.); neptune26ljs@naver.com (J.-S.L.); lastdohee@gmail.com (H.-J.I.); khs910707@hanmail.net (H.-S.K.); sky161300@naver.com (S.-B.L.); 2 TKM-Based Herbal Drug Research Group, Korea Institute of Oriental Medicine, Daejeon 34054, Korea; hwlee@kiom.re.kr

**Keywords:** atopic dermatitis, herbal medicine, inflammation, NC/Nga murine model

## Abstract

We evaluated the anti-atopic dermatitis (AD) effect of *Atofreellage* (AF), a herbal formula composed of 10 medicinal plants. AD was induced on the dorsal skin areas of NC/Nga mice (male, seven weeks old) by daily application of 2,4-dinitrochlorobenzene (DNCB) for five weeks. After three weeks of DNCB application, 200 μL of AF (0, 25, 50 or 100 mg/mL) was applied to the skin lesions. Histological findings, blood cell populations, serum levels of immunoglobulin E (IgE), histamine, pro-inflammatory cytokines, and inflammatory signaling in the skin tissue, and T-helper cell type 2 (Th_2_)-related cytokines in splenocytes were analyzed. Histopathological findings showed AF treatment notably attenuated the thickness of dorsal skin, and eosinophil infiltration. AF treatment (especially 100 mg/mL) also demonstrably ameliorated the blood cell population abnormalities, as the notable elevation of serum concentrations of IgE, histamine, TNF-α, IL-6 and IL-1β were remarkably normalized by AF treatment. Western blot analysis evidenced the apparent normalization of inflammatory signals (ERK, p38 MAP kinase, JNK, and NF-κB) in the skin tissue. Additionally, AF treatment notably attenuated the activation of Th_2_-dominant cytokines (IL-13, IL-4, and IL-5) in Con A-treated splenocytes in an *ex vivo* assay. In conclusion, this study provides experimental evidence for the clinical relevance of *Atofreellage*.

## 1. Introduction

Atopic dermatitis (AD), a critical medical issue, is a chronically relapsing inflammatory skin disease. Currently, the global prevalence of AD is approximately 1% to 20% [1]. The morbidity rate of AD has continuously increased, possibly due to industrialization and urbanization [2]. Moreover, both infants and children are the main subjects suffering from AD, which occasionally distorts healthy development in both physical and emotional terms [3].

The main symptoms of AD are as follows: dry and eczematous skin, erythematous papules, and severe pruritus [4]. Although the pathology of AD remains unclear, it is thought to involve hypersensitivity type 1 immune reactions [5]. The development of AD is mainly attributed to skin barrier defects and the deregulation of both T-helper cell type 1 (Th_1_) and T-helper cell type 2 (Th_2_) from the immune system [6]. Additionally, environmental factors such as allergens and microbes appear to play critical roles in the AD [7].

The complex and unexplored etiology of AD has made the development of therapeutics difficult. In general, for the past few decades anti-histamine agents, immunosuppressive agents, moisture care therapy, steroid ointments, or localized immunoregulatory agents have been used for treating AD [8,9]. Whole body steroid treatment is often chosen for severe cases of AD [10]. These therapeutics, however, do not radically treat AD, but rather, reduce AD-related symptoms and are frequently associated with side effects or relapse [11]. Accordingly, the above therapies have limitations regarding their use in treating patients with AD, and development of novel remedies as well as establishment of the disease etiology are required [12].

Various herbal plants have traditionally, been used for patients with skin dermatitis. Some herbal plants such as *Illicium verum* and *Catalpa ovata* stem bark have a potent effect on AD in animal models [13,14]. To invent an herbal medicine-derived remedy for AD, we surveyed the list of medicinal plants traditionally used in clinical practice and conducted an experimental screening using the rat-derived basophilic leukemia cell line RBL-2H3, focusing on the regulation of AD-related actions. We finally selected different plants and made up a formula, named “*Atofreellage*”, consisting of an equal weight of 10 herbal plants: *Rhus javanica* Linne, *Kochia scoparia* Schrader, *Cnidium monieri* Cuss, *Houttuynia cordata* Thunberg, *Schizonepeta tenuifolia* Briquet, *Sophora flavescens* Aiton, *Rheum palmatum* Linne, *Lithospermum erythrorhizon* Siebold et Zuccarini, *Terminalia chebula* Retzins, and *Trichosanthes kirilowii* Maximowicz. *Atofreellage* has been applied to AD skin lesions as a homemade “bath preparation” or “lotion type” treatment at Daejeon Oriental Hospital since 2014.

In the present study, we aimed to identify the anti-AD effects of *Atofreellage* and investigate its underlying pharmacological mechanisms using a 2,4-dinitrochlorobenzene (DNCB)-induced AD model in NC/Nga mice.

## 2. Results

### 2.1. Chemical Constitution Analysis of Atofreellage

**Figure 1:**
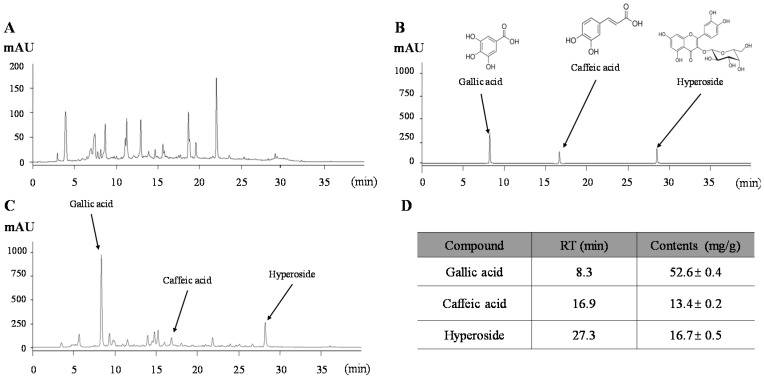
Fingerprint of *Atofreellage*. *Atofreellage* and its reference compounds were subjected to HPLC analysis. Histograms of *Atofreellage* (**A**,**C**) and reference compounds (**B**) and the quantitative analysis of *Atofreellage* (**D**) are presented.

The HPLC-based fingerprint of *Atofreellage* was conducted under the UV wavelength of 340 nm (Figure 1A), and quantitative analysis was conducted for three compounds. The retention times of gallic acid, caffeic acid and hyperoside were 8.3 min, 16.9 min, and 27.3 min, respectively (Figure 1B,C). Gallic acid at approximately 52.6 ± 0.4 mg/g was the most abundant component in *Atofreellage*, followed by hyperoside (16.7 ± 0.5 mg/g) and caffeic acid (13.4 ± 0.2 mg/g), (Figure 1D).

### 2.2. Effects on the Histopathological Analysis

**Figure 2:**
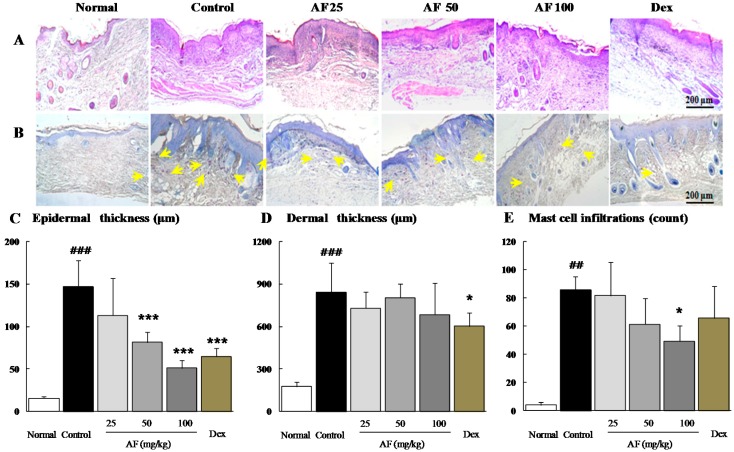
Histopathological findings. Dorsal skin lesions of NC/Nga mice were stained with H & E (**A**) and toluidine blue staining (**B**). All images were analyzed under 100× magnification. The skin thicknesses of epidermal (**C**) and dermal (**D**) tissues, and infiltration mast cells (**E**) were analyzed. The data are expressed as the mean ± SD (*n* = 8). Arrows indicated the infiltration of basophils. ^##^ *p* < 0.01 and ^###^ *p* < 0.001, compared with the normal group; * *p* < 0.05 and *** *p* < 0.001, compared with the control group.

Notably, H & E staining revealed the typical features of inflammatory cell infiltration into the skin, which was markedly ameliorated by *Atofreellage* treatment (Figure 2A). In addition, DNCB treatment drastically increased the thickness of both the epidermal and dermal tissues by approximately 9.3-fold and 4.8-fold, whereas *Atofreellage* treatment significantly ameliorated these changes compared with the control group, especially for epidermal tissue (*p* < 0.001 for 50 and 100 mg/mL *Atofreellage*, Figure 2A,C). The change in dermal thickness did not reach statistical significance (Figure 2D). Moreover, toluidine blue staining indicated the prominent number of mast cells in the dermal area, whereas *Atofreellage* treatment considerably decreased the number of mast cells (Figure 2B). The mast cell infiltrations were drastically increased as 21.4-fold in control group as compared with normal group, whereas *Atofreellage* treatment significantly reduced them as compared with control group (Figure 2E).

Dexamethasone treatment also remarkably ameliorated the above alterations, similar to *Atofreellage* treatment.

### 2.3. Effects on the Peripheral Blood Cell Populations

DNCB treatment significantly increased total leukocyte counts in the peripheral blood by 1.5-fold compared with the normal group, and the numbers of subpopulations, especially neutrophils, eosinophils, and basophils were increased by approximately 2.3-, 12.0-, and 5.0-fold compared with the normal group. These alterations were significantly attenuated by *Atofreellage* treatment compared with the control group (*p* < 0.05 for 100 mg/mL *Atofreellage* for neutrophils and eosinophils, *p* < 0.05 or <0.01 for 25 or 100 mg/mL *Atofreellage*, respectively, for basophils; Table 1). In the dexamethasone treatment group, the altered numbers of neutrophils, eosinophils, and basophils were significantly normalized.

**Table 1:** Effects on the peripheral blood cell population.

Categories	Normal	Control	*Atofreellage* (200 μL/Head)			Dexamethasone 3 mg/kg
			25 mg/mL	50 mg/mL	100 mg/mL	
WBC (k/μL)	2.34 ± 0.65	3.51 ± 0.75 ^#^	3.58 ± 1.04	2.75 ± 0.62	2.94 ± 0.61	2.80 ± 0.16
Lymphocyte (k/μL)	1.68 ± 0.31	1.97 ± 0.57	2.19 ± 0.44	1.83 ± 0.35	2.21 ± 0.42	2.1 ± 0.22
Monocyte (k/μL)	0.06 ± 0.01	0.07 ± 0.02	0.07 ± 0.02	0.07 ± 0.01	0.06 ± 0.02	0.07 ± 0.01
Neutrophil (k/μL)	0.59 ± 0.11	1.33 ± 0.20 ^##^	1.18 ± 0.15	0.78 ± 0.24	0.59 ± 0.16 *	0.56 ± 0.13 *
Eosinophil (k/μL)	0.01 ± 0.01	0.12 ± 0.03 ^###^	0.08 ± 0.06	0.08 ± 0.06	0.08 ± 0.03 *	0.04 ± 0.04 **
Basophil (k/μL)	0.01 ± 0.00	0.05 ± 0.01 ^##^	0.03 ± 0.02 *	0.03 ± 0.02 **	0.02 ± 0.02 **	0.02 ± 0.02 **

The data are expressed as the mean ± SD (*n* = 8). ^#^ *p* < 0.05, ^##^ *p* < 0.01 and ^###^ *p* < 0.001 compared with the normal group; * p < 0.05 and ** p < 0.01 compared with the control group.

### 2.4. Effects on Serum IgE and Histamine

**Figure 3:**
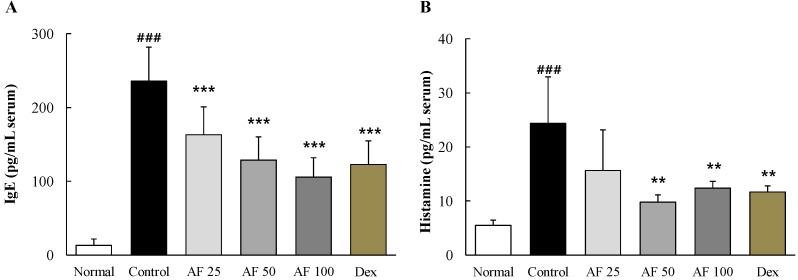
IgE and histamine in serum. Serum levels of IgE (**A**) and histamine (**B**) were measured using ELISA kit. The data are expressed as the mean ± SD (*n* = 8). ^###^ *p* < 0.001, compared with the normal group; ** *p* < 0.01 and *** *p* < 0.001, compared with the control group.

DNCB treatment drastically elevated serum IgE levels by approximately 17.7-fold compared with the normal group, whereas *Atofreellage* treatment significantly decreased this abnormal elevation in a dose-dependent manner (*p* < 0.001 for 25 to 100 mg/mL *Atofreellage*, Figure 3A). Serum histamine levels were remarkably increased by approximately 4.4-fold in the DNCB-treated control group compared with the normal group, while *Atofreellage* treatment significantly attenuated those abnormalities (*p* < 0.01 for 50 and 100 mg/mL *Atofreellage*, Figure 3B). Treatment with dexamethasone significantly attenuated the changes in both serum IgE and histamine levels.

### 2.5. Effects on Serum Pro-Inflammatory Cytokines

**Figure 4:**
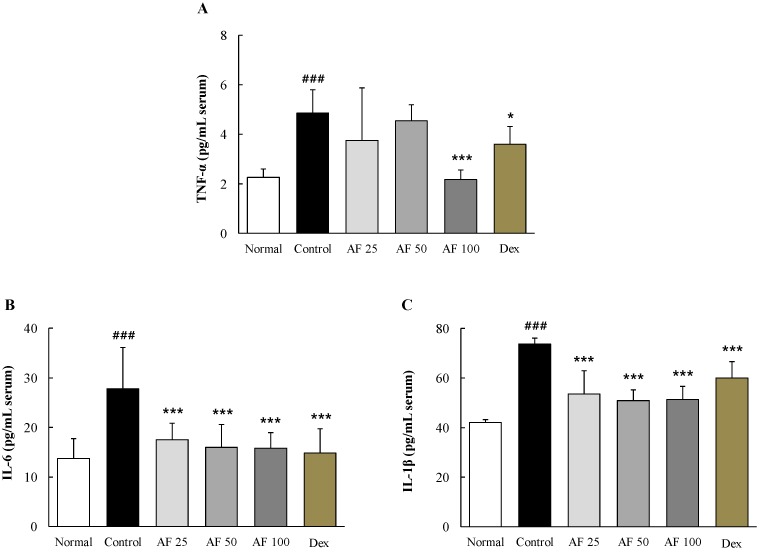
Pro-inflammatory cytokines in the serum. Serum levels of pro-inflammatory cytokines, including TNF-α (**A**); IL-6 (**B**); and IL-1β (**C**), were measured using ELISA kits. The data are expressed as the mean ± SD (*n* = 8). ^###^ *p* < 0.001, compared with the normal group; * *p* < 0.05 and *** *p* < 0.001, compared with the control group.

DNCB treatment markedly increased serum levels of three pro-inflammatory cytokines, TNF-α by 2.1-fold, IL-6 by 2.0-fold and IL-1β by approximately 2.1-fold, compared with the normal group. These alterations in serum cytokine levels were significantly attenuated by *Atofreellage* treatment compared with the control group, including the alterations in TNF-α (*p* <0.001 for 100 mg/mL *Atofreellage*) and in both IL-6 and IL-1β (*p* <0.001 for 25 to 100 mg/mL *Atofreellage*) (Figure 4A–C). Dexamethasone showed similar effects on the serum levels of TNF-α, IL-6 and IL-1β.

### 2.6. Effects on Inflammatory Signaling Pathways

**Figure 5:**
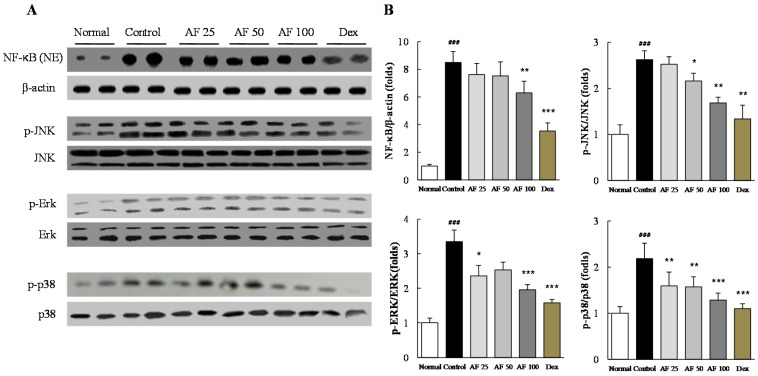
Western blot analysis of inflammatory signaling pathway components. Protein levels of NF-κB and MAP kinase sub-family members were determined by western blotting (**A**). Protein levels were quantified using ImageJ software (**B**). The data are expressed as the mean ± SD (*n* = 8). ^###^ *p* < 0.001 compared with the normal group; * *p* < 0.05, ** *p* < 0.01, and *** *p* < 0.001, compared with the control group.

DNCB treatment remarkably increased the protein levels of nuclear NF-κB in the skin tissues compared with those in the normal group, whereas *Atofreellage* treatment significantly reduced NF-κB activation compared with the control group (*p* < 0.01 for 100 mg/mL *Atofreellage*, Figure 5A,B). MAP kinase signaling molecules were considerably activated by DCNB treatment compared with the normal group, whereas *Atofreellage* treatment significantly attenuated the abnormally increased protein activities for p-JNK (*p* < 0.05 or < 0.01 for 50 and 100 mg/mL *Atofreellage*), p-ERK (*p* < 0.05 or < 0.001 for 25 and 100 mg/mL *Atofreellage*), and p38 MAP kinase (*p* < 0.01 or < 0.001 for 25 to 100 mg/mL *Atofreellage*) (Figure 5A,B). Treatment with dexamethasone also led to the normalization of both NF-κB and MAP kinase sub-family activation.

### 2.7. Effects on Th_2_ Cell-Related Cytokines in Primary Cultures of Splenocytes

**Figure 6:**
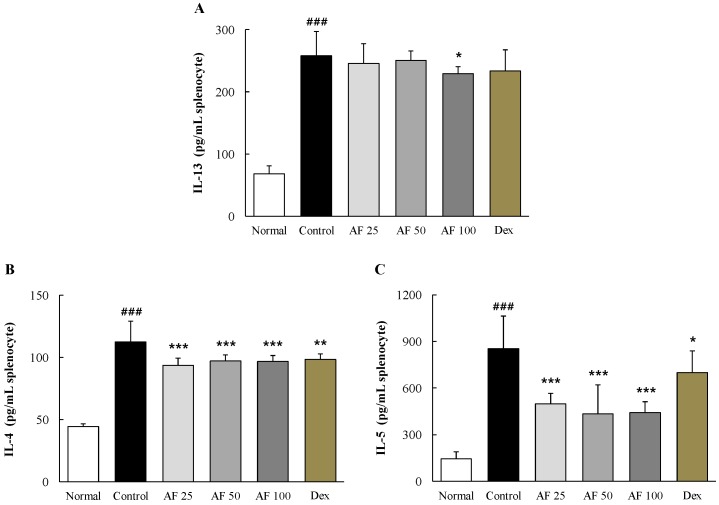
Th_2_-related cytokines in cultured splenocytes. The levels of IL-13 (**A**); IL-4 (**B**) and IL-5 (**C**) in culture media were measured using ELISA kits. The data are expressed the mean ± SD (*n* = 8). ^###^ *p* < 0.001, compared with the normal group; * *p* < 0.05 and *** *p* < 0.001, compared with the control group.

In *ex vivo* primary splenocytes stimulated by Con A, the control group showed considerably higher levels of Th_2_ cell-related cytokines, with increases of approximately 3.8-, 2.4-, and 5.9-fold for IL-13, IL-4, and IL-5, respectively, compared with the normal group. The *Atofreellage* treatment groups showed significant attenuation of those changes in the levels of IL-13 (*p* < 0.05 for 100 mg/mL *Atofreellage*, Figure 6A) and both IL-4 and IL-5 (*p* < 0.001 for 25 to 100 mg/mL *Atofreellage*, Figure 6B,C). Dexamethasone had positive effects on the levels of these cytokines.

## 3. Discussion

AD is a chronic inflammatory skin disease that occurs most frequently in both infants and children, and is increasing in prevalence [15]. Many groups have tried to identify therapeutic means to ameliorate AD, but limited success has been achieved to date, and there remains a great demand for novel AD therapeutics. The development of therapeutics has focused on the modulation of pathological mechanism(s) of the AD, likely including the inhibition of Th_2_ responses, the decrease of IgE production, and the production of anti-histamine effects [16,17]. Recently, medicinal plants have been widely adapted as an important resource in drug development for treating AD based on their long history of traditional clinical uses and relatively safe application [18].

In the present study, we aimed to explore the pharmacological effects of a drug mixture comprising multiple herbs called *Atofreellage*. We adapted a DNCB-induced AD model in NC/Nga mice; this model has been widely used for experimental studies due to its close resemblance to human AD [19]. Regarding the present AD remedies, applying drugs to the skin lesion is important to consider because the main complaints of AD include pruritus, dryness, and psoriasis on skin regions [20]. We therefore applied *Atofreellage* to the dorsal areas of AD-induced NC/Nga mice. As expected, the DNCB treatment induced thickening of the dermis and epidermis as well as the infiltration of inflamed cells on dermal areas of the skin lesion, which is a general feature of the histopathological characteristics in AD [21]. These histopathological alterations were significantly ameliorated by *Atofreellage* (Figure 2A–D).

Although the mechanistic details are not known, the abnormal accelerated production of IgE and antigen cross-linking of IgE on the receptors of mast cells and eosinophils followed by the eruption of histamine have been well known as the sequential events causing the symptoms of AD [5]. Accordingly, the elevated serum levels of IgE and histamine are a classical feature of AD; these are then considered as the main parameters to determine anti-atopic effects in many studies [22,23]. Several studies showed high levels of serum IgE and histamine in children with severe AD [24,25]. We observed that the dermal application of *Atofreellage* (especially 50 or 100 mg/mL) showed a similar efficacy as the oral administration of dexamethasone on the both IgE and histamine in the serum levels (Figure 3A,B). In the current study, we used as a positive drug dexamethasone, which is a strong immunosuppressant that has been used locally or systematically for severe cases of AD [26].

The imbalance between Th_1_ and Th_2_ in the development and progression of AD is well known; a Th_2_-dominant status stimulates the excessive production of IgE and the activation of mast cells [22]. Therefore, the modulation of Th_2_ cells has also been mainly targeted for AD remedies [27]. Th_2_-related cytokines, including IL-4, IL-5, and IL-13, can also cause excessive accumulation of mast cells, basophils, eosinophils and dendritic cells in skin lesions [5]. In the present study, the production of IL-4, IL-5, and IL-13 by splenocytes in *ex vivo* assays was notably modulated in *Atofreellage*-treated groups compared with the control group (Figure 6A–C). The mast cells have the same characteristics as basophils, and their filtration in dermal lesions was notably reduced by *Atofreellage* treatment, as shown with toluidine blue staining (Figure 2A). Mast cells and basophils release inflammatory chemicals, including histamine, while eosinophils mediate late-phase reactions such as destruction of the surrounding tissue at sites of the Th_2_-dominated inflammation [28].

The above findings were well supported by the effects of *Atofreellage* on the leukocyte cell number measurements in the peripheral blood levels. *Atofreellage* treatment significantly inhibited the increase of basophils and eosinophils in the peripheral blood (Table S1); the numbers of these two cell populations are frequently increased in patients suffering from AD [29]. AD is also a typical chronic relapsing inflammatory disease, especially in the case of localized skin lesions, and is an obstinate disease. Generally, pro-inflammatory cytokines, including TNF-α, IL-6, and IL-1β, are dominantly increased during the development or progression of AD [30]. In the current study, we found that the serum levels of TNF-α, IL-6, and IL-1β were markedly increased during DNCB-treated AD mode; however these increases were significantly ameliorated by *Atofreellage* treatment (Figure 4A–C).

Moreover, NF-κB and MAP kinase play critical roles in most inflammation by modulating the secretion of pro-inflammatory cytokines [30]. Previous studies documented that the suppression of MAP kinase activation led to reduction the number of mast cells, which are involved in the inflammatory response [26]. Based on the deep linkage between the inflammatory response during AD and MAP kinase signaling cascade molecules [31], thus we further explored the activities of JNK, ERK, and p38 as well as NF-κB in dermal samples. In our study, the excessively over-activated molecules due to DCNB application, but they were remarkably inhibited by *Atofreellage* treatment (Figure 5A,B). These results support the underlying mechanisms at least partially explaining the anti-atopic effect of *Atofreellage*.

*Atofreellage* was formulated based on extensive clinical experience and experimental screening for IL-4 and IL-13 inhibitory activity. We confirmed the reproducibility of *Atofreellage* using HPLC. The three most representative chemical compounds in *Atofreellage* are gallic acid, caffeic acid, and hyperoside (Figure 1A–D). In fact, among the herbs comprising *Atofreellage,* several herbs, including *R. javanica* Linne, *K. scoparia* Schrader, *C. monieri* Cuss, *H. cordata* Thunberg, *S. tenuifolia* Briquet, *S. flavescens* Aiton, *R. palmatum* Linne, *L. erythrorhizon* Siebold et Zuccarini, *T. chebula* Retzins, and *T. kirilowii* Maximowicz partially showed potent effects on AD [32,33,34,35,36,37]. Two main compounds, gallic acid and caffeic acid, also showed inhibitory actions on histamine release by mast cells and inflammatory reactions in an AD model [28,38]. Especially gallic acid not only substantially suppressed the IL-6, but also ameliorated the activation of MAP kinase signaling pathways, including p38 MAP kinase and JNK in the human basophilic KU812 cells [39]. Meanwhile, caffeic acid showed its anti-AD effects via reduction of histamine release in the compound 48/80-induced ICR mouse model [40]. A previous study also reported that the anti-AD effects of *Artemisia* capillaries, using the *Dermatophagoides farinae* ointment-induced NC/Nga mice model. Similarly to our study, *Artemisia* capillaries showed substantially the reduced plasma levels of both histamine and IgE [41], meanwhile this herb contains caffeic acid and hyperoside as main active compounds. Above studies support the pharmacological effects of *Atofreellage* against AD. The current multiple herb composition of *Atofreellage* would be disadvantageous in the process of developing an AD remedy due to difficulties in quality control and in the identification of active compounds [42]. The traditional clinical use of herbal medicine, however, generally involves multiple mixtures, which gives the advantage of safety. In addition, multiple herbal formulae exert multiple actions against multiple known or unknown molecular targets to control complex diseases such as AD [43]. *Atofreellage* has been developed based on the traditional use in clinics and experimental data. We confirmed the absence of cytotoxicity of *Atofreellage* at 400 mg/mL in RBL-2H3 cells, a rat-derived mast cell line. We showed herein the clinical relevance of *Atofreellage* as a skin application remedy and its underlying mechanisms corresponding to the pharmacological effects.

Taken together, our data provide experimental evidence of the pharmaceutical activity of *Atofreellage* for the treatment of AD. The underlying mechanisms corresponding to the anti-atopic effects of *Atofreellage* involve the regulation of IgE production, of histamine release via the modulation of Th_2_-dominant cytokines and anti-inflammatory activities in dermal lesions and whole blood.

## 4. Experimental Section

### 4.1. Chemicals

The following reagents were used: 2,4-dinitrochlorobenzene (DNCB; Sigma-Aldrich, St. Louis, MO, USA), dexamethasone (Sigma-Aldrich), acetone (Daejung, Gyonggi-do, Korea), olive oil (Sigma-Aldrich), toluidine blue (Sigma-Aldrich), hematoxylin (Acros Organics, Morris Plains, NJ, USA), eosin (Sigma-Aldrich), ethanol (Samchun, Gyeonggi-do, Korea), tris-glycine SDS buffer (LPS Solution, Daejeon, Korea), tris-glycine buffer (LPS Solution), polysorbate 20 (Tween 20,Duchefa Biochemie, Haarlem, The Netherlands), RIPA buffer solution (LPS Solution), formaldehyde solution (Samchun), and bovine serum albumin (Sigma-Aldrich).

### 4.2. Preparations of Atofreellage and Chemical Component Analysis

The 10 types of herbal plants composing *Atofreellage* (*R. javanica* Linne, *K. scoparia* Schrader, *C. monieri* Cuss, *H. cordata* Thunberg, *S. tenuifolia* Briquet, *S. flavescens* Aiton, *R. palmatum* Linne, *L. erythrorhizon* Siebold et Zuccarini, *T. chebula* Retzins, and *T. kirilowii* Maximowicz, Table S1) were purchased from the Jeong-Seong Oriental Pharmacy Store (Daejeon, Korea), and their identification was confirmed by a professional herbal pharmacist. *Atofreellage* was prepared as follows: briefly, 100 g each of the 10 fully dried individual herbs were mixed and boiled in 10 L of distilled water (DW) for 100 min at 100 °C using a high-speed automatic non-pressure earthen pot (Dae-Woong, Seoul, Korea), and the extraction procedure was then repeated with 5 L of water. *Atofreellage* was then centrifuged for 30 min at 1500× *g*, and the supernatant was lyophilized using a vacuum-freeze-drying system and stored at −70 °C. The final extraction yield was 6.5%. *Atofreellage* was dissolved in distilled water before use, and the remainder was stored at −70 °C for future use.

### 4.3. Obtaining the Fingerprint of Atofreellage

For reproducibility, *Atofreellage* was verified by producing its fingerprint using high-performance liquid chromatography (HPLC). Three reference compounds, gallic acid (*vs. T. chebula* Retzins), caffeic acid (*vs. S. flavescens* Aiton and *L. erythrorhizon* Siebold), and hyperoside (*vs. H. cordata* Thunberg), were used for additional quantitative analysis. The calibration curves of each chemical compound were obtained by assessing the peak areas at six concentrations in the range from 6.25 μg/mL to 200 μg/mL (serial dilution) for all reference compounds. The linearity of the peak area (y) *vs.* concentration (x, μg/mL) curve for each compound was used to calculate the content of the *Atofreellage* components (Figure 1A–D).

Quantitative analysis was performed using an 1100 series HPLC device (Agilent Technologies, Santa Clara, CA, USA) equipped with an autosampler (G11313A), column oven (GA1316A), binary pump (G1312), diode-array detector, and degasser (GA1379A). The analytical column was a Gemini C18 (4.6 mm × 250 mm; particle size, 5 μm; Phenomenex, Torrance, CA, USA) that was kept at 30 °C during the analysis. The mobile phase conditions contained 10% acetonitrile in water with 0.05% formic acid and 90% acetonitrile in water. The gradient flow was as follows: 0–30 min, 5%–95% B; 30–40 min, 30%–70% B; and 40–50 min, 80%–20%. The analysis was operated at a flow rate of 1.0 mL/min and detected at 254 and 340 nm. The injection volume was 10 μL. The data were acquired and processed using ChemStation software (Agilent Technologies).

### 4.4. Animal and Experiment Schedule

The design and performance of the animal experiment were approved by the Institutional Animal Care and Use Committee of Daejeon University (DJUARB2015-006), and conducted in accordance with the Policy on the Care and Use of Laboratory Animals. A total of forty-eight specific pathogen-free seven-week-old male NC/Nga (NC) mice were purchased from Dae-Han Bio Link (Choong-Book, Korea). All of the mice were acclimated for 1 week. After acclimation, all of the mice were randomly divided into the following six groups (*n* = 8 for each group): normal (vehicle without DNCB treatment), control (DNCB treatment without AF or Dex treatment), AF 25 (*Atofreellage* 25 mg/mL with DNCB treatment), AF 50 (*Atofreellage* 50 mg/mL with DNCB treatment), AF 100 (*Atofreellage* 100 mg/mL with DNCB treatment), and Dex (dexamethasone 3 mg/kg with DNCB treatment). To induce AD-like skin inflammation, DNCB was applied to the dorsal area of each animal, except for those of the normal group, according to a previously described method, with slight modifications [25]. DNCB (0.4% or 0.2%) was dissolved in an acetone and olive oil solution mixture (3:1) and was topically applied to mice daily for five weeks on the shaved dorsal skin (0.4% DNCB for three weeks and then 0.2% DNCB for the following two weeks). *Atofreellage* (200 μL of 0, 25, 50, or 100 mg/mL, topically applied to the dorsal skin) or dexamethasone (Dex; 3 mg/kg, orally) was given to the corresponding groups daily for 2 weeks, at which point the mice were treated with 0.2% DNCB (Figure S1).

On the final experimental day, all of the mice were euthanized under ether anesthesia after 12 h of fasting. After the collection of retro-orbital sinus blood for leukocyte profile analysis, whole blood was isolated from the abdominal vein using 1-mL syringes for peripheral blood cell counts and biochemical analyses. The spleens were immediately removed for *ex vivo* experiments, and the dorsal skin tissues were removed and immediately fixed in 10% formalin solution for histopathological examination or were stored at −70 °C for protein assays.

### 4.5. Histopathological Examination of AD-Like Skin Lesions

For the histopathological evaluation, freshly removed dorsal skin tissues were fixed in 10% neutral formalin and then subjected to an automatic tissue paraffin procedure using a programmed cascade. The paraffin-embedded samples were sectioned to a thickness of 4 μm and stained with hematoxylin and eosin (H & E) or toluidine blue. After H&E staining of the skin lesions, hyperplasia of the epidermal and dermal tissues was observed under a microscope (100×, Olympus IX71, Tokyo, Japan). On one photograph per sample, the lengths of epidermal and dermal tissues were measured in five different, randomly selected areas using Focus Pro histological analysis software (Focus, Daejeon, Korea). For the detection of mast cells or eosinophils in the dermal areas, toluidine blue-stained cells were observed under a visible light microscope (100×, Olympus IX71).

### 4.6. Analysis of Leukocytes in Peripheral Blood

Before whole blood collection on the final experimental day, approximately 60–80 μL of retro-orbital sinus blood was collected using a heparin-coated capillary tube. The numbers of total leukocytes, lymphocytes, monocytes, neutrophils, eosinophils, and basophils were counted using an automatic analyzer, CELL-DYNs 3200 (Abbott Laboratories, Santa Clara, CA, USA).

### 4.7. Measurement of total IgE and Histamine Concentrations in Serum

Under ether anesthesia conditions, whole blood was collected via the abdominal vein, and serum was obtained by centrifugation of the blood (3000× *g*, 15 min). The serum levels of total IgE and histamine were determined using enzyme-linked immunosorbent assay (ELISA) kits according to the manufacturers’ instructions (Cat. No. 555248, BioLegend, San Diego, CA, USA for IgE; KA1888, Abnova, Taipei City, Taiwan, for histamine).

### 4.8. Measurement of Pro-Inflammatory Cytokine Concentrations in Serum

The serum levels of three pro-inflammatory cytokines, tumor necrosis factor alpha (TNF-α), interleukin-1 beta (IL-1β), and interleukin-6 (IL-6), were measured using commercial ELISA kits (Biosource, Camarillo, CA, USA; R & D Systems, Minneapolis, MN, USA).

### 4.9. Western Blot Analysis

From the dorsal skin tissues, western blot analyses were performed to detect the expression of nuclear factor kappa B (NF-κB), c-Jun N-terminal kinase (JNK), phospho-JNK (p-JNK), extracellular signal-regulated kinase (ERK; p42/44), phospho-ERK (p-ERK; p-p42/44), p38 mitogen-activated protein (MAP) kinase (p38 MAP kinase), and phospho-p38 MAP kinase (p-p38 MAP kinase). The skin tissue samples were homogenized in radioimmunoprecipitation assay buffer (50 mM Tris-HCl, pH 7.4, 1% Nonidet P-40, 0.5% sodium deoxycholate, 150 mM NaCl) containing protease inhibitor cocktail (Roche, Indianapolis, IN, USA). To analyze NF-κB protein in the nucleus, the nuclear fraction was isolated using hypertonic buffer (100 mM Tris, pH 7.4, 2 mM Na_3_VO4, 100 mM NaCl, 1% Triton X-100, 1 mM EDTA, 10% glycerol, 1 mM EGTA, 0.1% SDS, 1 mM NaF, 0.5% deoxycholate, 20 mM Na_4_P_2_O_7_).

Skin protein extracts (30 μg of each sample) were separated by 10% sodium dodecyl sulfate-polyacrylamide gel electrophoresis (SDS-PAGE). After blocking membranes using 5% bovine serum albumin (Sigma-Aldrich) in 0.1% phosphate-buffered saline with Tween? 20 (PBST), western blotting was performed based on the manufacturer's instructions for the primary antibodies against NF-κB, JNK, p-JNK, ERK (p42/44), p-ERK (p-p42/44), p38 MAP kinase, and p-p38 MAP kinase (Thermo Scientific Co., San Jose, CA, USA). After incubation with primary antibodies and washing, the membranes were then incubated with secondary antibodies (rabbit or mouse) conjugated with peroxidase for 1 h, followed by washing with PBST. The signals were developed using a chemiluminescence detection buffer (ECL Femto Supersignal, Thermo Scientific Co.). The band intensity for all proteins was analyzed using ImageJ software (NIH, Rockville, MD, USA). β-actin was used as a reference protein.

### 4.10. Ex Vivo Experiment for the Determination of Th_2_ Cell-Related Cytokines

After the spleens were freshly removed from the mice of each experimental group, they were washed in cold PBS (10 mM, pH 7.4) twice. For primary cell culture, the splenocytes were isolated and then seeded into 6-well microplates at a density of 2 × 106/mL. The primary splenocytes were incubated in DMEM (Dulbecco’s modified Eagle’s medium, Lonza, Basel, Switzerland) containing 10% fetal bovine serum (FBS) overnight, and the medium was then changed. The splenocytes were stimulated with concanavalin A (ConA; 5 μg/mL, Sigma, San Diego, CA, USA) for 72 h. Protein levels of IL-13, IL-4, and IL-5 in the supernatants were determined using ELISA kits (from R & D Systems, for IL-13 and IL-4; BioLegend for IL-5).

### 4.11. Statistical Analysis

All data are expressed as the mean ± standard deviation (SD). One-way analysis of variance (ANOVA) was performed using SPSS 20.0 software (IBM SPSS Inc., Chicago, IL, USA), followed by Duncan’s multiple comparison tests to compare between groups. Differences with *p* < 0.05 were considered statistically significant.

## Supplementary Materials

Supplementary materials can be accessed at: http://www.mdpi.com/1420-3049/21/1/35/s1.
