# Supplementary material for: A Herbal Formula, Atofreellage, Ameliorates Atopic Dermatitis-Like Skin Lesions in an NC/Nga Mouse Model
Source: Molecules. 2015 Dec 25;21(1):35. doi: 10.3390/molecules21010035 (PMC6273903; doi:10.3390/molecules21010035)
Supplement: Supplementary file 1 [file molecules-21-00035-s001.pdf]

Supplementary Materials: An Herbal Formula, *Atofreellage*, Ameliorates Atopic Dermatitis-Like Skin Lesions in an NC/Nga Mouse Model

Won-Yong Kim [1], Hyeong-Geug Kim [1], Hye-Won Lee [2], Jin-Seok Lee [1], Hwi-Jin Im [1], Hyo-Seon Kim [1], Sung-Bae Lee [1] and Chang-Gue Son [1,*]

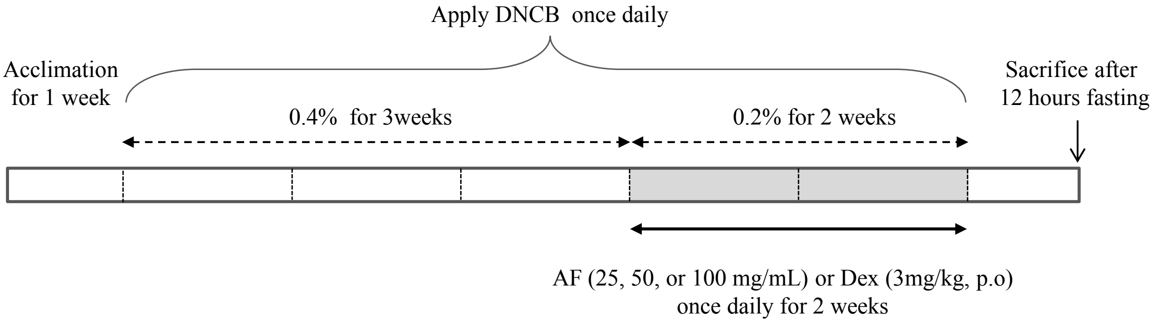

Figure S1. Experimental scheme. After 1 week acclimation, all mice were randomly divided into the following six groups (n = 8): normal (vehicle without DNCB treatment), control (DNCB treatment without AF or Dex treatment), AF 25 (Atofreellage 25 mg/mL with DNCB treatment), AF 50 (Atofreellage 50 mg/mL with DNCB treatment), AF 100 (Atofreellage 100 mg/mL with DNCB treatment), and Dex (dexamethasone 3 mg/kg with DNCB treatment). To induce AD-like skin inflammation, DNCB applied to mice daily for five weeks on the shaved dorsal skin, except for those of the normal group (0.4% DNCB for three weeks and then 0.2% DNCB for the following two weeks). Atofreellage (200 μL of 0, 25, 50, or 100 mg/mL, topically applied to the dorsal skin) or dexamethasone (Dex; 3 mg/kg, orally) was given to the corresponding groups daily for 2 weeks, at which point the mice were treated with 0.2% DNCB.

Table S1. Compositional herbs of *Atofreellage*

Herbal Name	Scientific Name	Chinese Name	Relative Amounts
Galla Rhois	*Rhus javanica* Linne	Wubeizi	1
Kochiae Fructus	*Kochia scoparia* Schrader	Difuzi	1
Cnidi Fructus	*Cnidium monieri* Cuss	Shengchuangzi	1
Houttuyniae Herba	*Houttuynia cordata* Thunberg	Yuxingcao	1
Schizonepetae Spica	*Schizonepeta tenuifolia* Briquet	Jingjie	1
Sophorae Radix	*Sophora flavescens* Aiton	Kushen	1
Rhei Rhizoma	*Rheum palmatum* Linne	Dahuang	1
Lithospermi Radix	*Lithospermum erythrorhizon* Siebold et Zuccarini	Zicao	1
Terminaliae Fructus	*Terminalia chebula* Retzins	Jiazi	1
Trichosanthis Radix	*Trichosanthes kirilowii* Maximowicz	Tianhuafen	1

Total 1kg (each 100 g of fully dried 10 herbs) was mixed and boiled in 10 L of distilled water for 100 min at 100 °C, and then centrifuged for 30 min at 1500× *g*. The supernatant was lyophilized using a vacuum-freeze-drying system and obtained final extraction (yield 6.5%).
